# MEDIATING ROLE OF REST–ACTIVITY RHYTHM ON THE RELATIONSHIP BETWEEN DEPRESSIVE SYMPTOM AND COGNITION IN OLDER ADULTS

**DOI:** 10.1093/geroni/igad104.3108

**Published:** 2023-12-21

**Authors:** Meina Zhang, Chooza Moon, Karin Hoth

**Affiliations:** University of Iowa, Iowa City, Iowa, United States; University of Iowa, Iowa City, Iowa, United States; University of Iowa, Iowa City, Iowa, United States

## Abstract

Depressive symptoms and cognitive impairments are common problems in older adults. Rest-Activity Rhythm (RAR) encompasses sleep, physical activity, and rest in 24 hours. Often, individuals with depressive symptoms present altered RAR. The purpose of the study is to investigate the mediating role of RAR on the relationship between depressive symptoms and cognition in older adults using path analysis. Sixty community-dwelling older adults wore the actigraphy for two weeks (mean age ± SD=70 ±7.3, female n =32(53%)). We assessed RAR using nonparametric RAR metrics, such as interdaily stability (IS) (the similarity of one 24-hour cycle to the next), intradaily variability (IV) (the fragmentation of RAR within 24-hour periods), and relative amplitude (RA) (physical activity difference between the least active 5 hours and the most active 10 hours period). Participants completed 90 minutes’ neuropsychological assessments (11 tests) across five domains to assess cognition and the Center for Epidemiologic Studies Depression scale to assess depressive symptoms. After adjusting for age, gender, and years of education, greater depressive symptoms were directly associated with reduced cognition (β= -0.406, P= 0.0001) as well as reduced IS (β= -26.9, P= 0.022). We also found direct positive relationships between IS (β=6.68, P=0.013), IV (β= 3.36, P=0.017), RA (β=3.42, P=0.015) and visuospatial memory, as well as IS (β=1.98, P=0.005), IV (β=3.77, P=0.004) and psychomotor speed. The indirect effect of depressive symptoms on cognition through RAR was not significant . Further research should be undertaken using a large, diverse sample, and prospective design.

